# Gallstone ileus presenting as intestinal obstruction in the elderly: Two case reports and literature review

**DOI:** 10.1016/j.ijscr.2025.111924

**Published:** 2025-09-08

**Authors:** Hazem Alouani, Mohamed Mahdi Trabelsi, Salsabil Nasri, Amine Ben Safta, Hichem Jerraya, Ramzi Nouira

**Affiliations:** a Department of General Surgery B, Charles Nicole's Hospital, Tunis, Tunisia

**Keywords:** Bilio-digestive fistula, Case report, Elderly, Enterolithotomy, Gallstone ileus, Intestinal obstruction

## Abstract

**Introduction and importance:**

Gallstone ileus is a rare and potentially life-threatening complication of chronic cholecystitis in the elderly. It results from the passage of a large gallstone through a bilio-digestive fistula into the gastrointestinal tract, causing mechanical obstruction. Early diagnosis and surgical intervention are crucial to reducing associated morbidity and mortality.

**Case presentation:**

We report two cases of gallstone ileus. Case 1: A 76-year-old man presented with vomiting, abdominal pain, and bowel obstruction. CT scan revealed pneumobilia and an obstructing gallstone in the distal ileum. A 3 cm stone was extracted via enterotomy, and a loop ileostomy was performed due to poor bowel viability. Case 2: A 65-year-old hypertensive woman presented with acute intestinal obstruction and hypovolemic shock. CT imaging showed a gallstone in the distal ileum. Enterolithotomy was performed initially, and a second surgery three months later included cholecystectomy and fistula repair.

**Clinical discussion:**

Gallstone ileus accounts for 1–4 % of intestinal obstructions, more common in the elderly. Rigler's triad is diagnostic but only seen in one-third of patients. CT imaging is the gold standard. Treatment typically involves enterolithotomy, with cholecystectomy and fistula repair deferred in high-risk patients.

**Conclusion:**

Gallstone ileus should be suspected in elderly patients with signs of obstruction and pneumobilia. CT imaging is essential for diagnosis. Individualized surgical strategies optimize outcomes.

## Highlights

• Gallstone ileus is a rare complication of chronic cholecystitis.
• It primarily affects elderly patients and should be suspected in small bowel obstruction with pneumobilia.
• Enterolithotomy is the primary treatment.
• Delayed cholecystectomy and fistula repair may be necessary in unstable patients.
• Loop ileostomy can be considered when bowel viability is compromised.

## 1 Introduction

Gallstone ileus is an uncommon yet serious complication of chronic gallbladder disease, representing 1–4 % of all mechanical bowel obstructions and up to 25 % in the elderly. It arises from chronic cholecystitis, leading to the formation of a bilio-digestive fistula through which large gallstones migrate into the intestinal tract. Obstruction usually occurs at the terminal ileum due to its narrow lumen. Risk factors include advanced age, female gender, repeated episodes of inflammation, and comorbidities such as cardiovascular disease. Diagnosis can be challenging due to the non-specific presentation and delayed onset. Rigler's triad—pneumobilia, ectopic gallstone, and bowel obstruction—is diagnostic but only present in a minority of cases. CT imaging offers the highest sensitivity.

Management of gallstone ileus remains controversial, particularly regarding one-stage vs. two-stage procedures. Enterolithotomy alone is favored in unstable patients, while cholecystectomy and fistula repair may be performed later.

We report Two cases of gallstone ileus in two elderly patients. These cases have been reported in line with the SCARE checklist [1].

## 2 Case report

### 2.1 Case 1

**Fig. 1:**
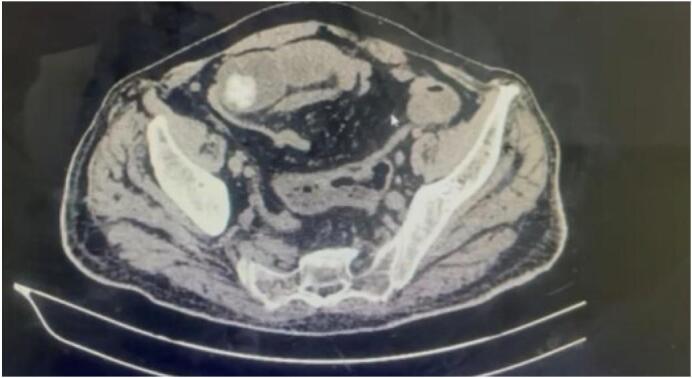
CT scan showing gallstone in terminal ileum.

**Fig. 2:**
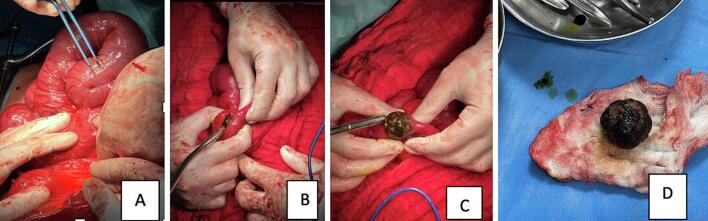
Intraoperative images of distended bowel and gallstone extraction.

A 76-year-old male with no significant past medical history presented after 7 days of vomiting, abdominal pain, and no bowel movements. He was afebrile and hemodynamically stable. Abdominal exam showed distension and diffuse tenderness. Labs revealed leukocytosis (17,000/mm^3^), CRP 175 mg/L, and renal insufficiency. He was classified ASA III. CT scan showed pneumobilia and a 3 cm obstructing gallstone in the distal ileum (Fig. 1). Intraoperatively, a distended small bowel and a fibrotic, atrophic gallbladder were noted. The gallstone was extracted through enterotomy (Fig. 2). Due to signs of bowel ischemia, a loop ileostomy was performed. No fistula was identified. The patient recovered without complications. The patient underwent uneventful ileostomy closure six weeks later and remained asymptomatic during a three-month follow-up period.

### 2.2 Case 2

**Fig. 3:**
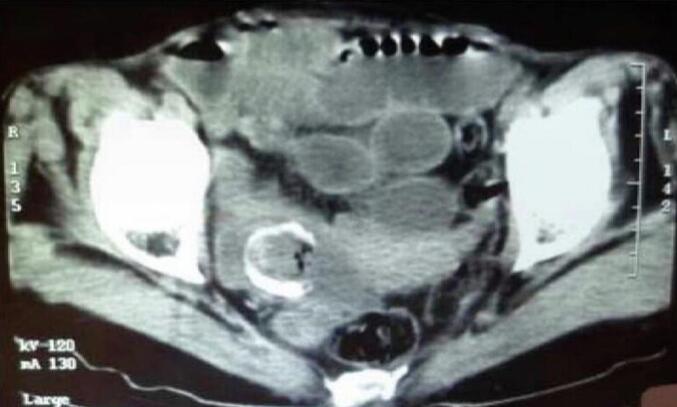
CT scan showing impacted gallstone in ileum (second case).

**Fig. 4:**
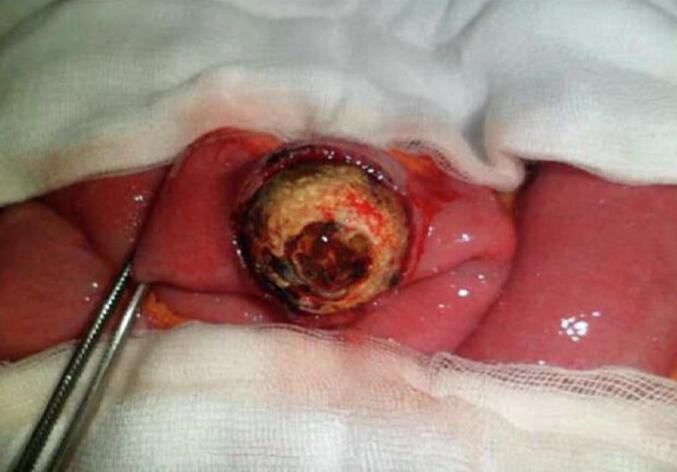
Enterotomy and stone removal (second case).

**Fig. 5:**
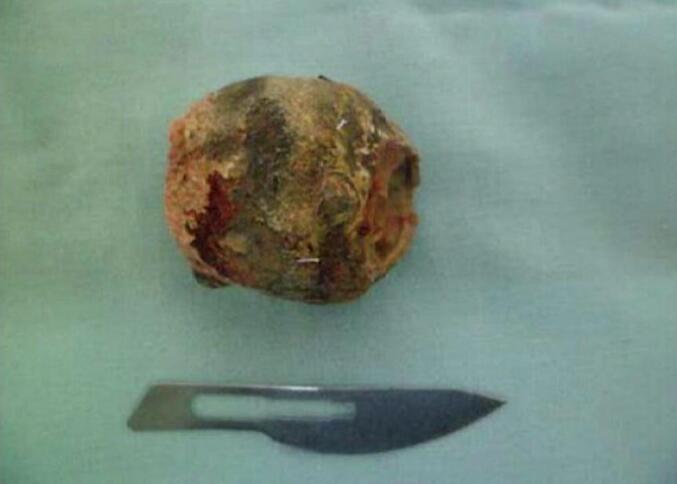
Extracted gallstone (3 cm).

A 65-year-old hypertensive woman with Raynaud syndrome presented with acute intestinal obstruction, dehydration, and shock (BP 100/60 mmHg, HR 120). Labs showed leukocytosis, elevated CRP, and pre-renal azotemia. CT confirmed pneumobilia and a gallstone in the terminal ileum (Fig. 3). ASA score was IV. An emergency enterolithotomy was performed (Fig. 4, Fig. 5). Intraoperatively, a bilio-digestive fistula was identified but not treated due to hemodynamic instability requiring vasopressors and ICU care. Three months later, she underwent elective cholecystectomy and closure of a cholecysto-duodenal fistula. She remained asymptomatic at 6-month follow-up.

## 3 Discussion

Gallstone ileus is an uncommon but significant complication of chronic cholecystitis, accounting for 1–4 % of mechanical bowel obstructions and complicating 0.3–0.5 % of cholecystitis cases [2]. Its incidence rises markedly in individuals over 65 years of age [3]. Patho physiologically, the condition results from a mechanical intestinal obstruction due to the endoluminal migration of a gallstone (typically larger than 2 cm). This migration occurs either through a bilio-digestive fistula formed secondary to recurrent episodes of acute cholecystitis and *peri* vesicular inflammation or, less commonly, as a postoperative complication following cholecystectomy [4].

The diagnosis is frequently delayed, contributing to elevated rates of morbidity and mortality [5]. It is based on Rigler's triad—pneumobilia, an ectopic gallstone, and features of bowel obstruction—though this triad is present in only 30–35 % of cases [6]. A 2020 systematic review by Ploneda-Valencia et al. analyzed over 100 cases of gallstone ileus and emphasized that early CT imaging significantly improved diagnostic rates and reduced time to intervention, directly impacting outcomes [7].

CT imaging is the preferred diagnostic modality, enabling identification of the transition point—most commonly located in the terminal ileum—and exclusion of additional stones. In 90 % of cases, the gallstone becomes impacted in the terminal ileum, followed by the colon in 7 % and the duodenum in 3 %, the latter presenting as Bouveret's syndrome [6].

Management focuses on resolving the mechanical obstruction. While spontaneous passage of the stone is rare, two principal surgical approaches exist: isolated enterolithotomy or enterolithotomy combined with cholecystectomy and fistula repair. In a retrospective review of 100 patients, Reisner and Cohen [8] reported a higher mortality (16.9 %) with the one-stage procedure compared to 11.7 % in enterolithotomy alone. This has led many to advocate for staged management in elderly or high-risk patients. A meta-analysis by Halabi et al. found no significant difference in mortality between one-stage and two-stage procedures, though morbidity was higher in combined surgeries, especially among elderly or hemodynamically unstable patients [9]. The combined approach is often deemed unnecessary in the absence of recurrent symptoms [10]. Recurrence after isolated enterolithotomy is rare (<5 %), and many fistulas close spontaneously. [11].

Our two cases demonstrate the adaptability of management based on intraoperative findings and patient condition. These approaches align with current trends favoring individualized management strategies based on intraoperative findings and patient risk stratification. Loop ileostomy was necessary in case 1 due to compromised viability, while a staged approach allowed safe delayed repair in case 2. Both patients recovered fully.

## 4 Conclusion

Gallstone ileus remains a rare but critical diagnosis in elderly patients with small bowel obstruction. Early CT imaging enables timely detection. Management must be individualized, considering bowel viability and hemodynamic status. Our two cases demonstrate the importance of surgical flexibility, from enterolithotomy with protective stoma to staged definitive surgery, in optimizing patient outcomes.

## Author contribution

**Hazem Alouani:** Data curation, Writing – original draft preparation **Mohamed Mahdi Trabelsi:** Conceptualization, methodology **Salsabil Nasri:** Visualization, Investigation **Amine Ben Safta:** Investigation **Hichem Jerraya:** Writing – Reviewing and Editing **Ramzi Nouira:** Supervision.

## Patients' perspectives

The male patient from Case 1 stated during follow-up: “*I'm grateful the doctors acted fast. I never imagined a gallstone could block my intestines*.”

The female patient from Case 2 shared: “*It was frightening, especially waking up in the ICU. But the second surgery really gave me peace of mind*.”

## Consent

Written informed consent was obtained from the patient for publication of this case. A copy of the written consent is available for review by the Editor-in-Chief of this journal on request.

## Ethical approval

This is a case report. Therefore, it didn't require ethical approval.

## Guarantor

Hazem Alouani.

## Research registration number

Not applicable.

## Author agreement statement

We the undersigned declare that this manuscript is original, has not been published before and is not currently being considered for publication elsewhere. We confirm that the manuscript has been read and approved by all named authors and that there are no other persons who satisfied the criteria for authorship but are not listed. We further confirm that the order of authors listed in the manuscript has been approved by all of us. We understand that the Corresponding Author is the sole contact for the Editorial process. He is responsible for communicating with the other authors about progress, submissions of revisions and final approval of proofs.

## Funding

The study did not receive any grant from funding agencies in the public, commercial or not-for profit sectors.

## Conflict of interest statement

None.
